# *RBFOX1* and *RBFOX3* Mutations in Rolandic Epilepsy

**DOI:** 10.1371/journal.pone.0073323

**Published:** 2013-09-06

**Authors:** Dennis Lal, Eva M. Reinthaler, Janine Altmüller, Mohammad R. Toliat, Holger Thiele, Peter Nürnberg, Holger Lerche, Andreas Hahn, Rikke S. Møller, Hiltrud Muhle, Thomas Sander, Fritz Zimprich, Bernd A. Neubauer

**Affiliations:** 1 Cologne Center for Genomics, University of Cologne, Cologne, Germany; 2 Cologne Excellence Cluster on Cellular Stress Responses in Aging-Associated Diseases (CECAD), University of Cologne, Cologne, Germany; 3 Department of Neuropediatrics, University Medical Clinic Giessen, Giessen, Germany; 4 Department of Neurology, Medical University of Vienna, Vienna, Austria; 5 Center for Molecular Medicine Cologne (CMMC), University of Cologne, Cologne, Germany; 6 Department of Neurology and Epileptology, Hertie Institute of Clinical Brain Research, Eberhard-Karls University, Tuebingen, Germany; 7 Danish Epilepsy Centre, Dianalund, Denmark; 8 Department of Neuropediatrics, University Medical Center Schleswig-Holstein, Christian-Albrechts University, Kiel, Germany; Innsbruck Medical University, Austria

## Abstract

Partial deletions of the gene encoding the neuronal splicing regulator *RBFOX1* have been reported in a range of neurodevelopmental diseases, including idiopathic generalized epilepsy. The RBFOX1 protein and its homologues (RBFOX2 and RBFOX3) regulate alternative splicing of many neuronal transcripts involved in the homeostatic control of neuronal excitability. In this study, we explored if structural microdeletions and exonic sequence variations in *RBFOX1, RBFOX2, RBFOX3* confer susceptibility to rolandic epilepsy (RE), a common idiopathic focal childhood epilepsy. By high-density SNP array screening of 289 unrelated RE patients, we identified two hemizygous deletions, a 365 kb deletion affecting two untranslated 5′-terminal exons of *RBFOX1* and a 43 kb deletion spanning exon 3 of *RBFOX3*. Exome sequencing of 242 RE patients revealed two novel probably deleterious variants in *RBFOX1,* a frameshift mutation (p.A233Vfs*74) and a hexanucleotide deletion (p.A299_A300del), and a novel nonsense mutation in *RBFOX3* (p.Y287*). Although the three variants were inherited from unaffected parents, they were present in all family members exhibiting the RE trait clinically or electroencephalographically with only one exception. In contrast, no deleterious mutations of *RBFOX1* and *RBFOX3* were found in the exomes of 6503 non-RE subjects deposited in the Exome Variant Server database. The observed *RBFOX3* exon 3 deletion and nonsense mutation suggest that *RBFOX3* represents a novel risk factor for RE, indicating that exon deletions and truncating mutations of *RBFOX1* and *RBFOX3* contribute to the genetic variance of partial and generalized idiopathic epilepsy syndromes.

## Introduction

Rolandic epilepsy (RE), or benign epilepsy with centrotemporal spikes (BECTS), is one of the most common epilepsy syndromes of childhood, comprising about 15% of epilepsies in children under the age of 16 years [1]. Age of onset ranges from years 3 to 13 and peaks between 8–9. Characteristic features are 1.) a somatosensory onset with unilateral paresthesias involving the tongue, lips, gums, and inner cheeks 2.) unilateral, tonic, clonic or tonic-clonic convulsions involving the face, lips, tongue as well as the pharyngeal and laryngeal muscles, causing 3.) speech arrest and drooling due to sialorrhea and saliva pooling. At this stage the seizure may end, or it may develop into a generalized tonic clonic seizure. Nocturnal seizures, the most frequent variant of this syndrome, frequently become generalized. The electroencephalographic hallmark, a prerequisite of diagnosis, are blunt high-voltage characteristically shaped centrotemporal spikes (CTS), often followed by slow waves [2], [3]. Based on a number of family studies it is generally assumed that, like in all other common idiopathic epilepsies a multifactorial mode of inheritance appears most likely [2], [4], [5]. The most recent family study on RE reported an increased rate of RE, febrile seizures, and an “epilepsy aphasia spectrum disorder” in relatives of children with RE [6]. To date, a number of loci or genes have been linked to various forms of idiopathic focal epilepsies or the EEG endophenotype of centrotemporal spikes (CTS). Linkage was reported to markers on 15q13.2 and to 16p12-11.2 [7], [8]. But no causative gene has been reported at either loci yet. In a small number of cases variants and mutations in *KCNQ2* and *KCNQ3* were found to be associated with RE and the respective EEG trait [9]. In addition, mutations in *SRPX2* in two families with mental retardation, severe language dysfunction and rolandic seizures have been reported [10]. By genome-wide linkage analysis RE and the CTS trait have been associated with the elongator protein complex 4 [11]. Furthermore, in a girl with early-onset epileptic encephalopathy and CTS a *de novo GRIN2A* mutation was identified [12]. However, all these findings in single or few patients or small families still lack replication.

Rare copy number abnormalities of *RBFOX1* have also been associated with mental retardation in comorbidity with and without seizures, attention deficit disorder and autism [13], [14], [15], [16]. Recently, we have shown in 1408 idiopathic generalized epilepsy (IGE) patients and 2256 population controls that deletions affecting 5′ located exons of *RBFOX1* are significantly enriched in IGE cases compared to population matched controls [17]. The *RBFOX* genes (*RBFOX1*: chr16∶6069132-7763340, NM_001142333; *RBFOX2*:, chr22∶36134783-36424585, NM_001082578, and *RBFOX3*: chr17∶77085427-77512230; NM_001082575) encode neuron-specific splicing factors predicted to regulate neuronal splicing networks. Several epilepsy candidate genes are downstream targets of Rbfox proteins (*FLNA, SLC1A3, DCX, GABRB3, GAD2, KCNQ2, SCN8A, SLC12A5, SV2B, SYN1*) and their regulation of expression and splicing has been demonstrated [18], [19]. FOX family members regulate splicing of the other FOX members and autoregulate themselves [20], [21].

The present candidate gene analysis tested whether (i) deletions in *RBFOX1, RBFOX2* and *RBFOX3* might increase risk of RE and (ii) exonic mutations affecting the protein structure occur more frequently in RE compared to control subjects.

## Methods

The institutional review board of the University Giessen, Germany specifically approved this study. Registration number: No 03/11. Written informed consent was obtained from all subjects or their legal guardians according to study protocols approved by the institutional ethical review board of the University Giessen, Germany under the title “genomic variation in patients with idiopathic epilepsy”.

### Diagnostic Criteria

Diagnosis of RE was performed according to the International Classification of Seizures and Epilepsies as described [3]. Sleep activation, characteristic shape, and classification by two independent individuals were required for classification of the EEG trait. Electrical Status Epilepticus in slow sleep (ESES) was diagnosed if prolonged generalized discharges of CTS dominated sleep EEG recordings [22], [23], [24]. Atypical benign partial epilepsy of childhood (ARE) was diagnosed employing the following criteria: Characteristic EEG trait of CTS, however, with trains of continuous generalized nocturnal discharges as a prerequisite of diagnosis in all ABPE cases. In addition at least one of the following two features needed to be present: (1) seizures compatible with BECTS plus one or more additive seizure types like astatic seizures, atypical absences (“dreamy states”) or myoclonic seizures as reported. (2) seizures compatible with BECTS plus a significant mental handicap, and/or severe developmental speech disorder [25], [26], [27]. If a child had a seizure symptomatology compatible with BECTS but had prolonged generalized discharges of CTS during sleep EEG, without any additional seizure types and a normal global and speech development it was diagnosed as BECTS.

### Patient Cohort

#### Families

The investigated cohort consisted of 98 index cases selected from 98 multiplex families with at least two affected siblings. In 96 families at least one of the affected probands suffered from RE or ARE, the second affected sibling presented either with RE, ARE or the EEG trait only. In two families all affected children did not suffer from seizures, but displayed the EEG trait only. Of all 98 index patients tested, 78 presented with RE, 15 with ARE, 3 with ESES, and 2 with CTS only.

#### Sporadic cases

191 non-familial cases were included into the study cohort. Of these patients 153 suffered from classic RE, 11 from ESES, 26 from ARE and 1 with CTS only.

### Genotyping and Copy Number Variation Detection

Whole blood DNA from the patients was genotyped for using the Infinium OmniExpressExome BeadChip (Illumina Inc., San Diego, CA) according to the manufacture’s protocol. Briefly, 200 ng of DNA were amplified, biotin labeled, and hybridized to the microarray. CNV calls were generated with the PennCNV software [28], using the log R ratio (LRR) and B allele frequency (BAF) for 730.525 probes designed for the genotyping array. CNV analysis was restricted to microdeletions covered by at least 20 probes and spanning 40 kb or more in size. To exclude technical artifacts, all potential microdeletions were manually inspected for regional SNP heterozygosity state and log2 ratios of the signal intensities using the Illumina Genome Viewer (Illumina Inc., San Diego, CA). Only one *RBFOX1* deletion and one *RBFOX3* deletion could be confirmed by manual variant evaluation. Segregation in the families of the microdeletions was examined by real-time quantitative PCR using TaqMan CNV probes (*RBFOX1:* Hs04461212_cn; *RBFOX3:* Hs03975574_cn) (Life Technologies, Darmstadt, Germany).

### Exonic Sequence Analysis

Sequence analysis was performed using next generation sequencing techniques. In brief, DNA was fragmented using sonification technology (Covaris, Woburn, MA, USA) and fragments were end repaired and adaptor ligated. SeqCap EZ Human Exome Library® v2.0 (Roche NimbleGen, Madison, WI, USA) was used for enrichment and samples were analyzed on the Illumina HiSeq 2000® sequencer. For 242 patients exome data were generated which featured an average coverage >30x for 77% of the target sequences. Data were filtered using Illumina Realtime Analysis® (RTA) software v1.8 and mapped to the human genome reference build hg19 via the ELANDv2 alignment algorithm on a multinode compute cluster. PCR duplicates were excluded using CASAVA v1.8. Variant calling was performed by SAMtools (version 0.1.7) for InDel detection. Scripts developed in-house at the Cologne Center for Genomics (Cologne, Germany) were applied to detect protein changes, affected splice sites, and overlaps with known variants. In particular, variants were filtered for high-quality unknown variants in *RBFOX1*, *RBFOX2,* and *RBFOX3* by comparison to an in-house variation database, dbSNP build 137 (www.ncbi.nlm.nih.gov/projects/SNP/), 1000 Genomes database (www.1000genomes.org/), and the Exome Variant Server (http://evs.gs.washington.edu/EVS/). Variant validation and segregation analyses were performed by Sanger sequencing following standard protocols.

## Results

### Detection of *RBFOX1* and *RBFOX3* Microdeletions in RE Patients

**Figure 1:**
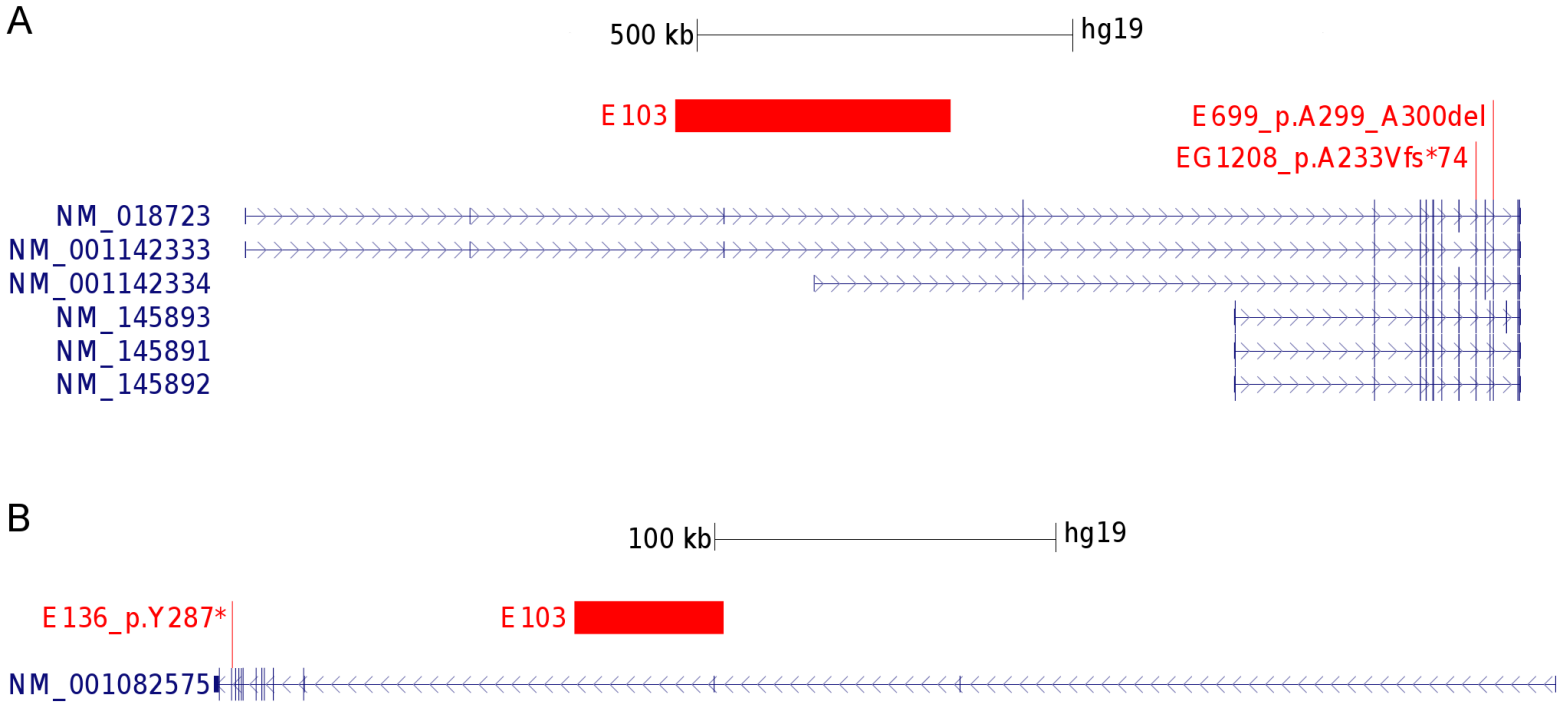
Overview of *RBFOX1* and *RBFOX3* affecting variants. Hg19 genomic localization and overview of known transcript variants. Red bars represent microdeletion size and location for the patient. Red dashes indicate the genomic location of single nucleotide variants. (A) Overview *RBFOX1* (B) *RBFOX3*.

In total, 289 RE patients were screened for copy number variations in *RBFOX1, RBFOX2,* and *RBFOX3* using the Infinium OmniExpressExome BeadChip® (Illumina Inc., San Diego, CA). We identified one RE patient among 289 (0.34%) with a hemizygous deletion in both *RBFOX1* and *RBFOX3*. A deletion of 365 kb was found to be located in the genomic region of *RBFOX1* affecting the untranslated 5′-terminal exons 3 and 1B (Fig. 1A, S1, exon annotation according to [17]) whereas a smaller deletion of 43 kb affected *RBFOX3* by removing exon 3 of the known isoform NM_001082575 along with flanking intronic sequences (Fig.1B, S1). The *RBFOX1* gene deletion affects the largest transcript variants 4–5 and 6 (NM_018723, NM_001142333, NM_001142334). No additional mutations were identified in the remaining undeleted *RBFOX1* and *RBFOX3* exonic sequences.

### Detection of Rare Exonic Variants in *RBFOX1*and *RBFOX*3

Mutational screening of *RBFOX1, RBFOX2, RBFOX3* in 242 RE patients did not reveal any mutation in *RBFOX2*, while a total of three rare mutations (1.2%) were identified in *RBFOX1* and *RBFOX3* (Fig. 1A,1B; Table 1). In contrast to the identified *RBFOX1* and *RBFOX3* microdeletions, all exonic variants were located near the 3′-terminal region of both genes. The identified exonic variants included two *RBFOX1* variants in exon 11 (c.690_696delGTATCCAins(GTATCCA)2; p.A233Vfs*74, NM_001142333) and exon 13 (c.893_898delCTGCCG, p.A299_A300del, NM_001142333), as well as a nonsense mutation in exon 13 of the *RBFOX3* gene (c.861C>A, p.Y287*, NM_001082575). The p.A299_A300del variant of patient E699 (Table 1, S1) deletes two out of three consecutive alanine residues which are conserved among mammals but not vertebrates (Fig. S2, S3).

**Table 1:** *RBFOX1and RBFOX3* variants and phenotype of index-patients.

Family	Index-patient	Patient	Variant	Epilepsy Syndrome	Diagnosis/seizure types	Comorbidity
**1**	x	E103	RBFOX1∶365kb Deletion, RBFOX3∶43 kb Deletion	RE	Nocturnal generalized tonic clonic seizures, Postictal speech arrest	None (normal global development, normal speech acquisition)
**1**		E103b	RBFOX1∶365 kb Deletion	CTS only	No seizures, EEG trait only	None (normal global development, normal speech acquisition)
**2**	x	EG1208	RBFOX1: p.A233Vfs*74	RE	Nocturnal rolandic seizures with postictal speech arrest	Initially delayed language development, later normal
**2**		EG1209	RBFOX1: p.A233Vfs*74	RE	Nocturnal and diurnal rolandic seizures with postictal speech arrest	Initially delayed language development, later normal
**3**	x	E699	RBFOX1: p.A299_A300del	RE	Nocturnal rolandic seizures with postictal speech arrest	None (normal global development, normal speech acquisition)
**3**		E699b	-	RE	Nocturnal rolandic seizures with postictal speech arrest	None (normal global development, normal speech acquisition)
**4**	x	E136	RBFOX3: p.Y287*	RE	Nocturnal generalized tonic clonic seizures with postictal speech arrest	Initially delayed language development, later normal
**4**		E679c	RBFOX3: p.Y287*	ESES	ESES without seizures	Moderate developmetal delay, delayed speech development, mild oral dyspraxia

Survey on *RBFOX1* and *RBFOX3* variants in patients. Seizure type and comorbidity overview of variant carrier. Abbreviations: RE = rolandic epilepsy; CTS = centrotemporal spikes; ESES = epileptic encephalopathy with status epilepticus during sleep.

To our knowledge nonsense mutations in *FOX* genes have not been described in the literature and are not found in any of the available databanks. They are absent from 6503 individuals whose exomes were deposited in the EVS database. Furthermore, no deleterious mutations have been identified in >450 exomes of our in-house database (various non epilepsy projects; about 80% Caucasian ancestry). In the truncated proteins the C-terminal fragments of RBFOX1 and RBFOX3 are affected which are critical for cassette-exon activation and repression [29] and the nuclear localization of the FOX proteins [21], [30], [31].

### Familial Segregation and Comorbidity Analysis

**Figure 2:**
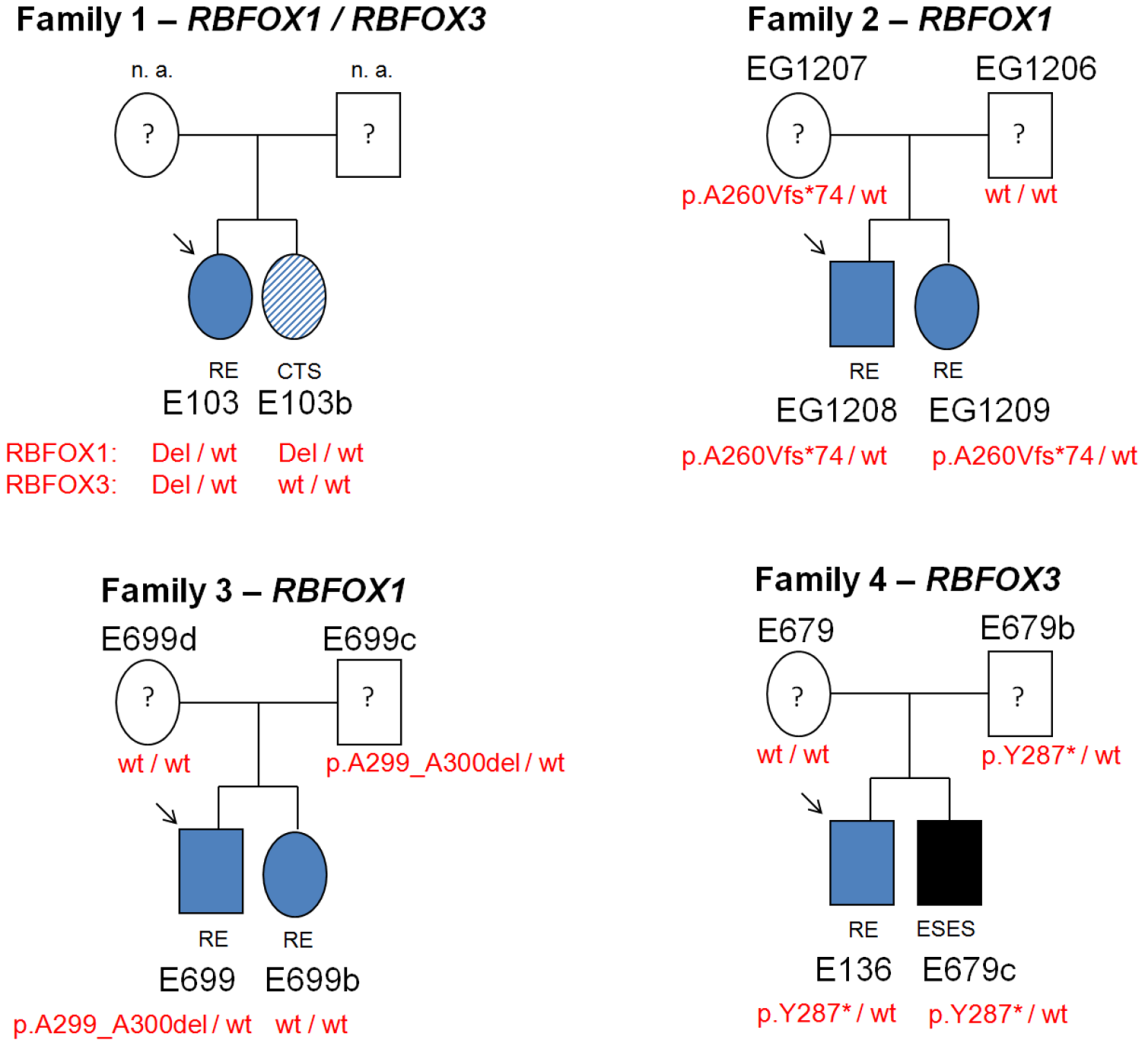
Segregation of *RBFOX1* and *RBFOX3* affecting variants. For three mutations for which DNA samples of family members were available, segregation analyses could be performed. The respective *RBFOX1* and *RBFOX3* truncating mutations co-segregated with a variable phenotype of either seizures or pathologic EEG patterns in most family members. Only a few individuals carried the respective familial mutation but did not present any clinical features, indicating incomplete penetrance of the mutations. However, subclinical phenotypes (e.g. EEG patterns) have not been investigated in these individuals (indicated by question mark). In family 3 the variant (deletion of two consecutive alanine residues at position 299–300 of RBFOX1) did not segregate with the epilepsy phenotype. Abbreviations: n.a = DNA was not available for testing; RE = rolandic epilepsy; CTS = centrotemporal spikes; ESES = encephalopathy with status epilepticus during sleep.

The segregation of *RBFOX1* and *RBFOX3* variants identified in the RE index-patients were tracked in four families (Fig. 2). Where testing was possible (n = 3), all variants identified were inherited, one maternally and two paternally. Five out of ten variant carriers were affected by RE, one by an encephalopathy with status epilepticus during sleep (ESES) and one by the RE-characteristic CTS EEG-trait only. The transmitting parents were all unaffected at the date of evaluation. Notably, we cannot rule out that the parents might have expressed the RE-characteristic CTS EEG-trait at younger age, considering its age-related expression with maximum manifestation during childhood. Six out of eight family members affected by either RE, CTS or ESES carried one exonic variant of *RBFOX1* or *RBFOX3* and one RE patient had a deletion in both genes (Fig. 2). The index patient in Family 1 had RE and exon-removing microdeletions in both *RBFOX1* and *RBFOX3* whereas her sister with the CTS EEG-trait alone carried only the *RBFOX1* microdeletion. In Families 1, 2 and 4, all affected family members carried a *RBFOX1* deletion or one of the truncating mutations. In family 3, the RBFOX1 p.A299_A300del deletion identified in the RE-index patient was not present in his RE-affected sister. The phenotypic RE features of the four variant carrying index cases did not differ from those RE patients lacking *RBFOX*1 and *RBFOX3* variants (Table 1). The ESES phenotype variant is generally assumed to represent the most severe expression of the RE CTS EEG-trait. A mild to moderate developmental speech delay, that frequently resolves later, is a known feature in many RE patients [32].

## Discussion

A previous study of 1408 unrelated individuals with idiopathic generalized epilepsy revealed exon-removing *RBFOX1* microdeletions in five patients, whereas none was found in 2256 ethnically matched controls [17]. Furthermore, rare copy number abnormalities of *RBFOX1* have been reported for patients with neurological diseases like epilepsy, mental retardation and autism [13], [14], [15], [16] strongly indicating that partial *RBFOX1* deletions are a recurrent risk factor of neurodevelopmental defects in human. The heterozygous *Rbfox1* knockout mouse model shows deregulated splicing which impacts genes involved in synaptic transmission and membrane excitability, leading to an increased susceptibility for seizure events. Notably, homo- and heterozygous *Rbfox1* knockouts display normal brain morphology [33]. Consistent with the splicing alterations in mice, RNA interference-mediated 50% knockdown of *RBFOX1* transcripts in human neurons changes the alternative splicing pattern and expression of primarily neuronal genes involved in synapse formation and function [19].

Our identification of a RE patient carrying a rare microdeletion affecting the 5′ part of *RBFOX1* replicates the association found in idiopathic epilepsy, but now in an entirely different, i.e. focal, epilepsy syndrome. Her sister, who also carried the deletion, exhibited the RE-specific EEG trait but did not suffer from overt seizures. In agreement with our previous IGE study, this indicates that *RBFOX1* microdeletions act as a susceptibility factor but not as a highly penetrant variant. The frequency of the *RBFOX1* deletion in the RE patient cohort (0.34%) is in the range of that observed for IGE (0.35%; [17]). The *RBFOX1* microdeletion presented here is located in the untranslated 5′-terminal region of the gene, like the previously reported microdeletions and translocations [13]–[17]. A female with autism carrying a 5′-terminal microdeletion of *RBFOX1* due to a *de novo* translocation t(15p;16p) displayed a significantly reduced *RBFOX1* mRNA expression in lymphocytes [14]. A second *FOX* microdeletion also detected in patient E103 is affecting the paralogous *RBFOX3* gene which also encodes the highly conserved RNA recognition motif (RRM) [20]. Interestingly, the sister of E103, who also carries the *RBFOX1* deletion but lacks the *RBFOX3* deletion, only expressed CTS but without having epileptic seizures. Due to the lack of further statistical and functional evidence, we can only hypothesize that both deletions affect neuronal splicing in Family 1 synergistically. Microdeletions in *RBFOX3* are extremely rare. Only two other exon-removing microdeletions have been noted in the Database of Genomic Variants (DGV, accessed 2/2013) in the general population. Being present in control individuals might indicate a variable expressivity of *RBFOX3* microdeletions or a lack of careful assessment of neuropsychiatric phenotypes in these probands. Both microdeletions do not delete exon 3 (NM_001082575) of *RBFOX3* as in our RE patient. It has been demonstrated that Rbfox3 regulates alternative splicing and nonsense mediated decay of *Rbfox2* mRNA [21]. This complex interplay of Fox family members has been further reported in the *Rbfox1* knockout mouse model where the loss of *Rbfox1* inhibits an upregulation of *Rbfox2* [33]. Mutational screening did not reveal any exonic mutation in *RBFOX2*, while three rare mutations have been identified (1.2%) in *RBFOX1* and *RBFOX3* together. The C-terminal fragment is critical for cassette-exon activation and repression [29] in *RBFOX1* as well as for nuclear localization for RBFOX1 [30], [31] and RBFOX3 [21]. These mechanisms are likely to be affected by the observed mutations. Furthermore, no frameshift nor nonsense mutations have been found in the exomes of 6503 control subjects reported in the ESV database. All individuals with an epileptic phenotype were carriers of the truncating mutations in family 1, 2 and 4. Only in family 3 the variant (deletion of two consecutive alanine residues at position 299–300 of *RBFOX1*) did not segregate with the epilepsy phenotype. This deleted sequence is conserved among mammals only, suggesting that most likely only truncating variants may be risk-conferring for RE. *In vitro* studies demonstrated that the C-terminal domain is critical for cellular localization in both *FOX* genes and also for targeted splicing in *RBFOX1* [21], [30], [31]. As a model for haploinsufficiency *in vitro* knock down of *RBFOX1* in primary human neural stem cells resulted in an altered expression and splicing of several epilepsy candidate genes (*FLNA, SLC1A3, DCX, GABRB3, GAD2, KCNQ2, SLC12A5, SV2B, SYN1*) [19]. Interestingly, variants and mutations in *KCNQ2* were recently found associated with RE [9].

In summary, our results strengthen the association of partial *RBFOX1* deletions in neurodevelopmental diseases and extend the *RBFOX1*-related phenotypic spectrum by RE. The present *RBFOX3* mutations highlight this neuronal gene as a plausible novel genetic risk factor of RE, and suggest that besides genomic microdeletions affecting the 5′-terminal exons, truncating mutations of *RBFOX1* and *RBFOX3* increase the risk of RE.

## Supporting Information

Figure S1 **Raw SNP intensity data of all samples carrying exon-disrupting microdeletions affecting the** ***RBFOX1*** **and** ***RBFOX3*** **genes.** Red frames represent the area of the observed microdeletions. Signal intensities of a SNP probe are represented by dots, one dot per each probe (Log R ratio track). A decline of neighboring probe signal intensities and B allele frequencies (B Allele Freq track) near 1 and 0 indicate a genomic deletion. The deletions have been visualized using the Illumina Genome Studio Software.
(DOC)

Figure S2 **UCSC Genome Browser** ***RBFOX1*** **transcript, common SNP and GERP conservation annotation tracks.** Top track: Highlighted in red, deleted nucleotides of patient E699. Middle track: The deleted sequence is abundant all six known *RBFOX1* transcripts. Lower track: No common SNP (≤1%) is annotated in dbSNP137 for the shown sequence interval. Bottom track: *RBFOX1* Genomic Evolutionary Rate Profiling (GERP) scores. The rejected substitutions score (RS) is based on an alignment of 35 mammal scores. A RS score threshold of 2 provides high sensitivity while still strongly enriching sequence conservation sites (http://www.genome.ucsc.edu). For the deleted sequence of *RBFOX1,* high and low RS scores are shown.
(DOC)

Figure S3 **Multiple Sequence Alignment** ***RBFOX1*** **variant A299_A300del (c.893_898delCTGCCG, p.A299_A300del, NM_001142333).** Multiple sequence alignments: The top line indicates the human amino acid sequence according to genome build hg19. Amino acids highlighted in red are hemizygously deleted in patient E699. Three alanine residues are conserved among mammalians but only one alanine residue in none mammalian vertebrates. Sequence annotations were taken from the UCSC Genome Browser (http://www.genome.ucsc.edu) and for multiple sequence alignments we used ClustalW (http://www.ebi.ac.uk/Tools/services/web_clustalw2/).
(DOC)

Table S1. ***RBFOX1*** **and** ***RBFOX3*** **exonic sequence variant.**
(DOC)
