# Correction: *RBFOX1* and *RBFOX3* Mutations in Rolandic Epilepsy

**DOI:** 10.1371/annotation/f6aed47b-9135-45f5-bfdd-f4ceb33c8561

**Published:** 2013-10-29

**Authors:** Dennis Lal, Eva M. Reinthaler, Janine Altmüller, Mohammad R. Toliat, EuroEPINOMICS Consortium, Holger Thiele, Peter Nürnberg, Holger Lerche, Andreas Hahn, Rikke S. Møller, Hiltrud Muhle, Thomas Sander, Fritz Zimprich, Bernd A. Neubauer

EuroEPINOMICS Consortium was not included in the author byline. It should be listed fifth. 

