# Supplementary material for: RBFOX1 and RBFOX3 Mutations in Rolandic Epilepsy
Source: PLoS One. 2013 Sep 6;8(9):e73323. doi: 10.1371/journal.pone.0073323 (PMC3765197; doi:10.1371/journal.pone.0073323)

**Figure S1 Raw SNP intensity data of all samples carrying exon-disrupting microdeletions affecting the *RBFOX1* and *RBFOX3* genes**

*RBFOX1* 365 kb deletion


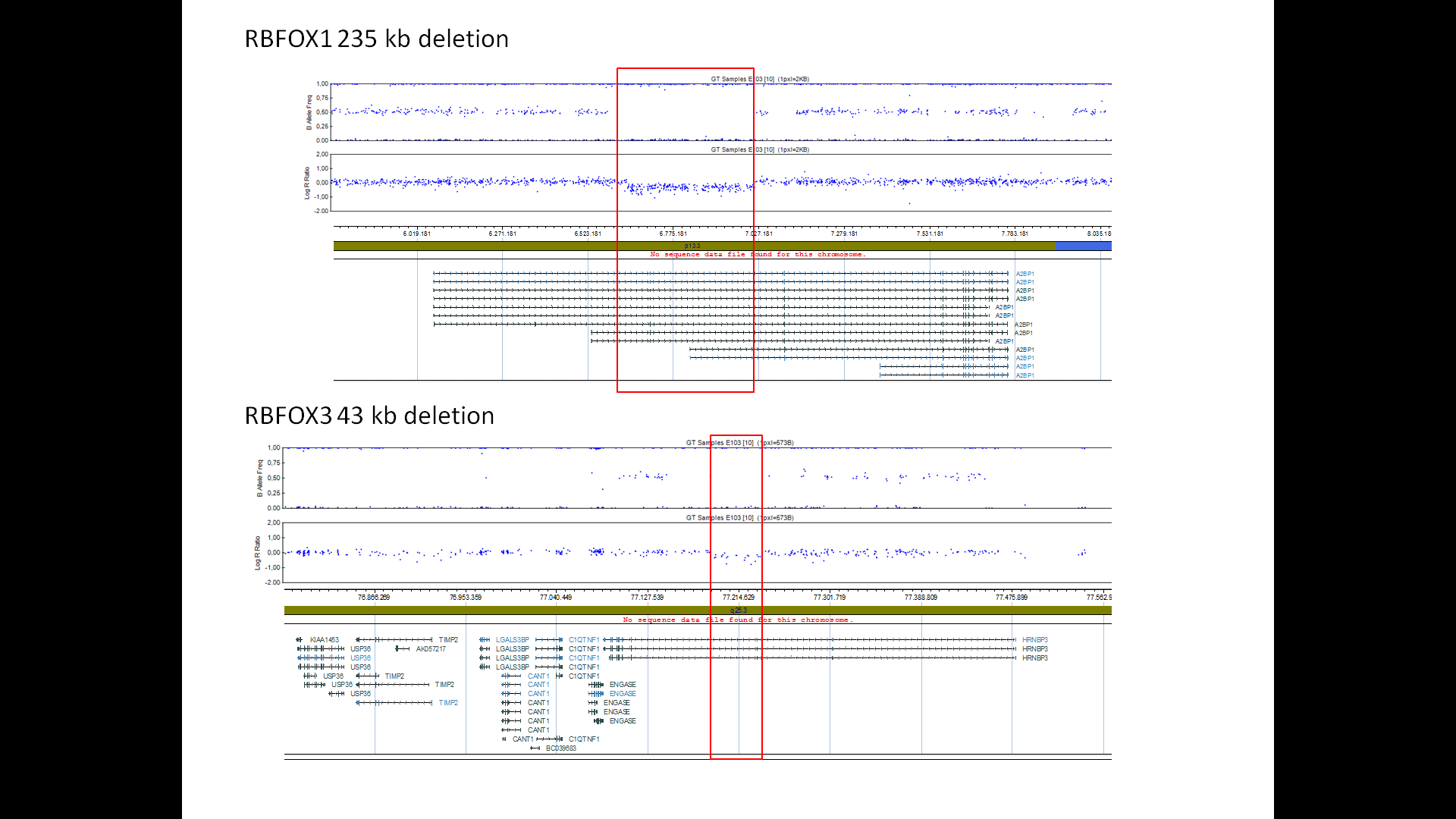


*RBFOX3* 43 kb deletion


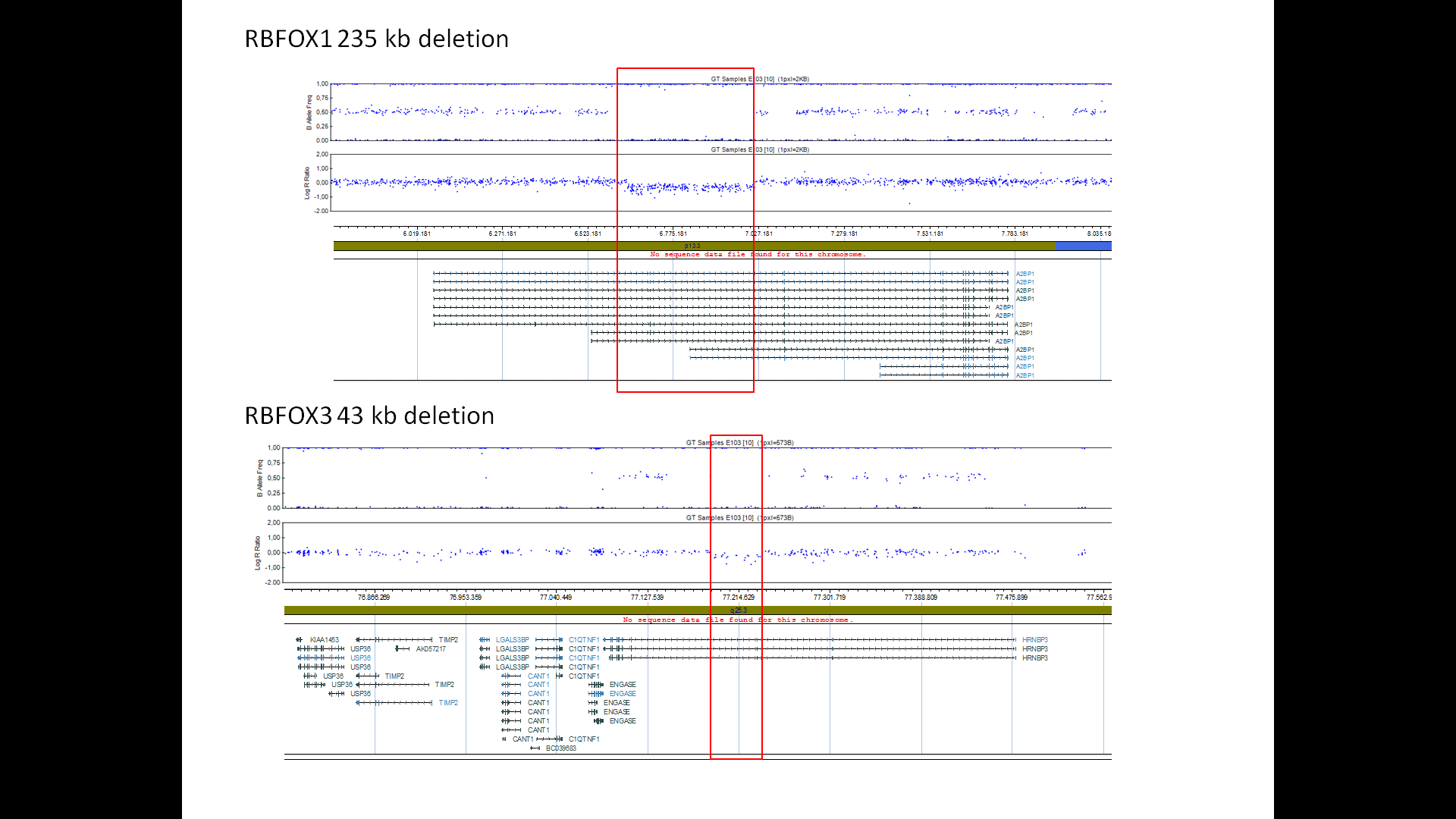

Supplement: Figure S1 — Raw SNP intensity data of all samples carrying exon-disrupting microdeletions affecting the RBFOX1 and RBFOX3 genes. Red frames represent the area of the observed microdeletions. Signal intensities of a SNP probe are represented by dots, one dot per each probe (Log R ratio track). A decline of neighboring probe signal intensities and B allele frequencies (B Allele Freq track) near 1 and 0 indicate a genomic deletion. The deletions have been visualized using the Illumina Genome Studio Software. (DOC) [file pone.0073323.s001.doc]
