# Supplementary figures and images for: RBFOX1 and RBFOX3 Mutations in Rolandic Epilepsy
Source: PLoS One. 2013 Sep 6;8(9):e73323. doi: 10.1371/journal.pone.0073323 (PMC3765197; doi:10.1371/journal.pone.0073323)

**Figure S2 Flanking sequence information for *RBFOX1* variant p.A299_A300del (c.893_898delCTGCCG, p.A299_A300del, NM_001142333)**


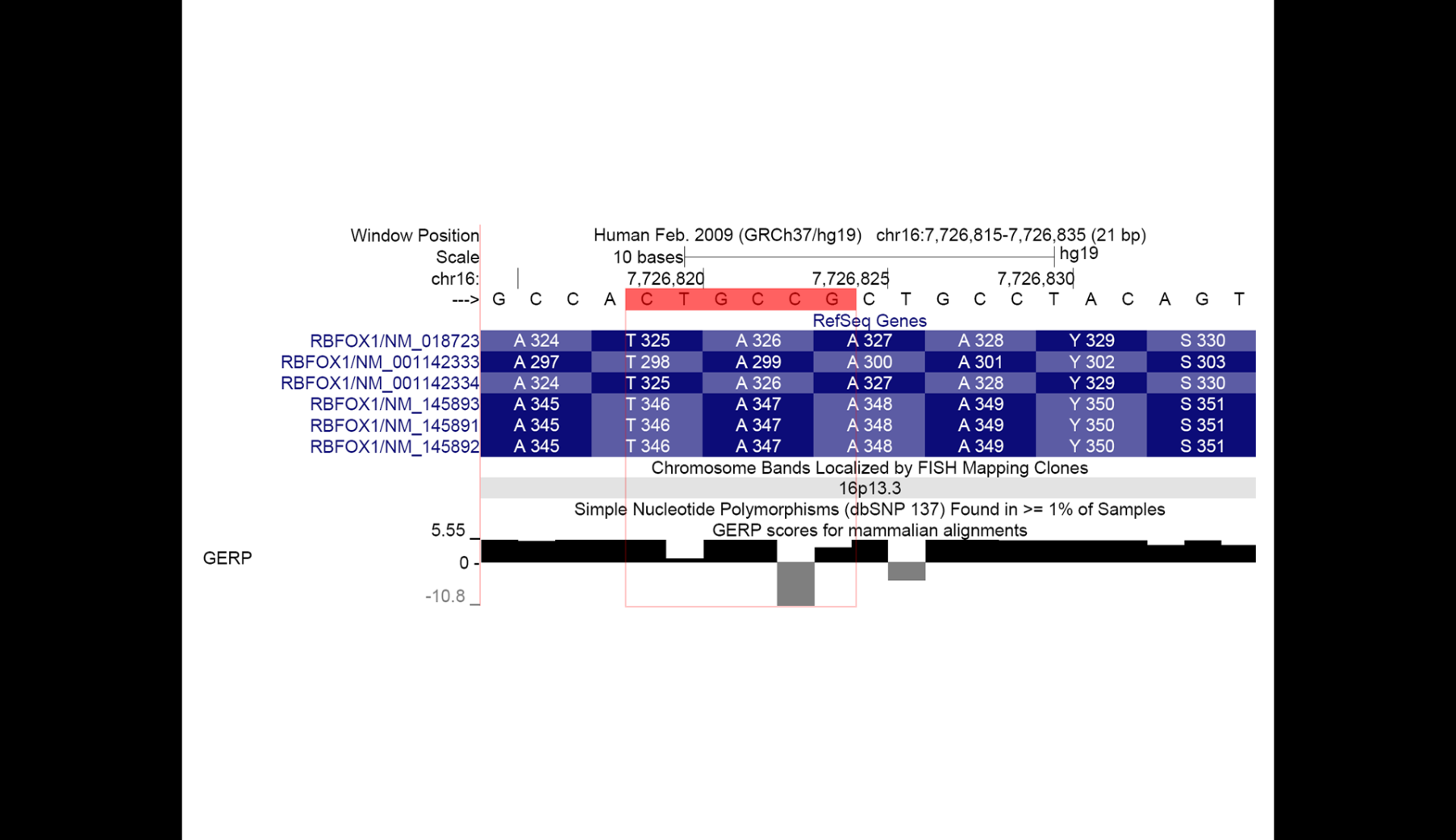

Supplement: Figure S2 — UCSC Genome Browser RBFOX1 transcript, common SNP and GERP conservation annotation tracks. Top track: Highlighted in red, deleted nucleotides of patient E699. Middle track: The deleted sequence is abundant all six known RBFOX1 transcripts. Lower track: No common SNP (≤1%) is annotated in dbSNP137 for the shown sequence interval. Bottom track: RBFOX1 Genomic Evolutionary Rate Profiling (GERP) scores. The rejected substitutions score (RS) is based on an alignment of 35 mammal scores. A RS score threshold of 2 provides high sensitivity while still strongly enriching sequence conservation sites (http://www.genome.ucsc.edu). For the deleted sequence of RBFOX1, high and low RS scores are shown. (DOC) [file pone.0073323.s002.doc]
