# Supplementary material for: RBFOX1 and RBFOX3 Mutations in Rolandic Epilepsy
Source: PLoS One. 2013 Sep 6;8(9):e73323. doi: 10.1371/journal.pone.0073323 (PMC3765197; doi:10.1371/journal.pone.0073323)
Supplement: Figure S3 — Multiple Sequence Alignment RBFOX1 variant A299_A300del (c.893_898delCTGCCG, p.A299_A300del, NM_001142333). Multiple sequence alignments: The top line indicates the human amino acid sequence according to genome build hg19. Amino acids highlighted in red are hemizygously deleted in patient E699. Three alanine residues are conserved among mammalians but only one alanine residue in none mammalian vertebrates. Sequence annotations were taken from the UCSC Genome Browser (http://www.genome.ucsc.edu) and for multiple sequence alignments we used ClustalW (http://www.ebi.ac.uk/Tools/services/web_clustalw2/). (DOC) [file pone.0073323.s003.doc]

**Figure S3** [**Multiple Sequence Alignment**](http://www.ebi.ac.uk/Tools/msa)***RBFOX1* variant A299_A300del**
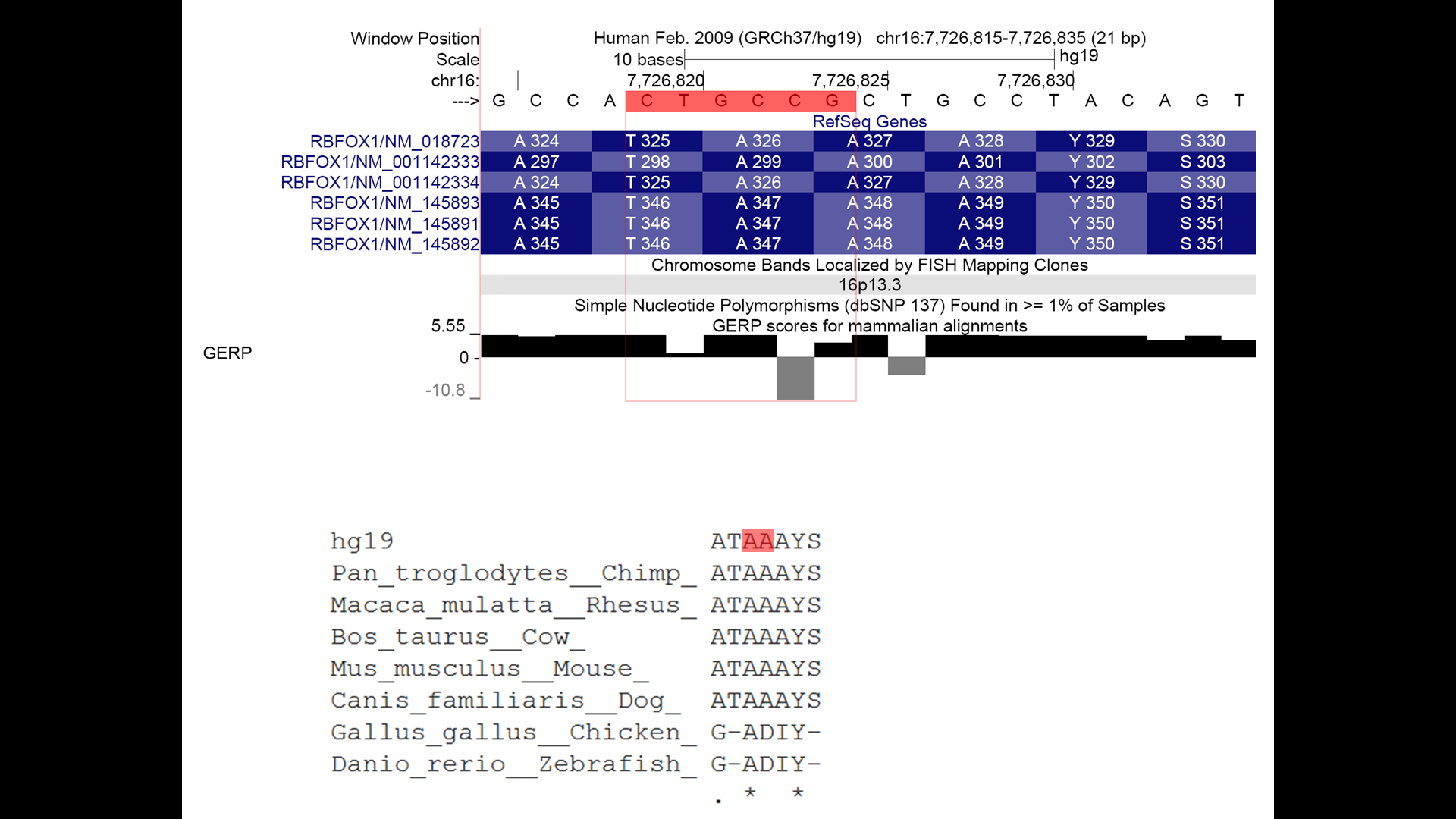
**(c.893_898delCTGCCG; NM_001142333)**
