# Supplementary material for: RBFOX1 and RBFOX3 Mutations in Rolandic Epilepsy
Source: PLoS One. 2013 Sep 6;8(9):e73323. doi: 10.1371/journal.pone.0073323 (PMC3765197; doi:10.1371/journal.pone.0073323)
Supplement: Table S1. — RBFOX1 and RBFOX3 exonic sequence variant. (DOC) [file pone.0073323.s004.doc]

**Table S1**. ***RBFOX1*and *RBFOX3* exonic sequence variants**

|  | **Start** | **End** | **Size** | **Ref** | **Allele** | **Type** | **HGNC** | **CCDS** | **RefSeq** | **MutPos** | **Consequence** | **MutCDNA** | **MutProt** | **Patient** |
| --- | --- | --- | --- | --- | --- | --- | --- | --- | --- | --- | --- | --- | --- | --- |
| 16 | 7703830 | 7703836 | 7 | GTATCCA | (GTATCCA)2 | INDEL | RBFOX1 | [CCDS55984.1](http://www.ncbi.nlm.nih.gov/CCDS/CcdsBrowse.cgi?REQUEST=CCDS&DATA=CCDS55984.1) | [NM_001142333.1](http://www.ncbi.nlm.nih.gov/nuccore/NM_001142333.1) | CDS.11 | FRAMESHIFT | c.690_696delGTATCCAins(GTATCCA)2 | p.A233Vfs*74 | EG1208 |
| 16 | 7703830 | 7703836 | 7 | GTATCCA | (GTATCCA)2 | INDEL | RBFOX1 | [CCDS55983.1](http://www.ncbi.nlm.nih.gov/CCDS/CcdsBrowse.cgi?REQUEST=CCDS&DATA=CCDS55983.1) | [NM_018723.3](http://www.ncbi.nlm.nih.gov/nuccore/NM_018723.3) | CDS.10 | FRAMESHIFT | c.771_777delGTATCCAins(GTATCCA)2 | p.A260Vfs*74 | EG1208 |
| 16 | 7703830 | 7703836 | 7 | GTATCCA | (GTATCCA)2 | INDEL | RBFOX1 | [CCDS55983.1](http://www.ncbi.nlm.nih.gov/CCDS/CcdsBrowse.cgi?REQUEST=CCDS&DATA=CCDS55983.1) | [NM_018723.3](http://www.ncbi.nlm.nih.gov/nuccore/NM_018723.3) | CDS.12 | FRAMESHIFT | c.771_777delGTATCCAins(GTATCCA)2 | p.A260Vfs*74 | EG1208 |
| 16 | 7703830 | 7703836 | 7 | GTATCCA | (GTATCCA)2 | INDEL | RBFOX1 | [CCDS10531.1](http://www.ncbi.nlm.nih.gov/CCDS/CcdsBrowse.cgi?REQUEST=CCDS&DATA=CCDS10531.1) | [NM_145891.2](http://www.ncbi.nlm.nih.gov/nuccore/NM_145891.2) | CDS.9 | FRAMESHIFT | c.831_837delGTATCCAins(GTATCCA)2 | p.A280Vfs*51 | EG1208 |
| 16 | 7703830 | 7703836 | 7 | GTATCCA | (GTATCCA)2 | INDEL | RBFOX1 | [CCDS45405.1](http://www.ncbi.nlm.nih.gov/CCDS/CcdsBrowse.cgi?REQUEST=CCDS&DATA=CCDS45405.1) | [NM_145892.2](http://www.ncbi.nlm.nih.gov/nuccore/NM_145892.2) | CDS.9 | FRAMESHIFT | c.831_837delGTATCCAins(GTATCCA)2 | p.A280Vfs*51 | EG1208 |
| 16 | 7703830 | 7703836 | 7 | GTATCCA | (GTATCCA)2 | INDEL | RBFOX1 | [CCDS10532.1](http://www.ncbi.nlm.nih.gov/CCDS/CcdsBrowse.cgi?REQUEST=CCDS&DATA=CCDS10532.1) | [NM_145893.2](http://www.ncbi.nlm.nih.gov/nuccore/NM_145893.2) | CDS.9 | FRAMESHIFT | c.831_837delGTATCCAins(GTATCCA)2 | p.A280Vfs*51 | EG1208 |
| 16 | 7726819 | 7726824 | 6 | CTGCCG | - | DEL | RBFOX1 | [CCDS10531.1](http://www.ncbi.nlm.nih.gov/CCDS/CcdsBrowse.cgi?REQUEST=CCDS&DATA=CCDS10531.1) | [NM_145891.2](http://www.ncbi.nlm.nih.gov/nuccore/NM_145891.2) | CDS.11 | DELETION | c.1037_1042delCTGCCG | p.A347_A348del | E699 |
| 16 | 7726819 | 7726824 | 6 | CTGCCG | - | DEL | RBFOX1 | [CCDS55984.1](http://www.ncbi.nlm.nih.gov/CCDS/CcdsBrowse.cgi?REQUEST=CCDS&DATA=CCDS55984.1) | [NM_001142333.1](http://www.ncbi.nlm.nih.gov/nuccore/NM_001142333.1) | CDS.13 | DELETION | c.893_898delCTGCCG | p.A299_A300del | E699 |
| 16 | 7726819 | 7726824 | 6 | CTGCCG | - | DEL | RBFOX1 | [CCDS55983.1](http://www.ncbi.nlm.nih.gov/CCDS/CcdsBrowse.cgi?REQUEST=CCDS&DATA=CCDS55983.1) | [NM_018723.3](http://www.ncbi.nlm.nih.gov/nuccore/NM_018723.3) | CDS.14 | DELETION | c.974_979delCTGCCG | p.A326_A327del | E699 |
| 16 | 7726819 | 7726824 | 6 | CTGCCG | - | DEL | RBFOX1 | [CCDS45405.1](http://www.ncbi.nlm.nih.gov/CCDS/CcdsBrowse.cgi?REQUEST=CCDS&DATA=CCDS45405.1) | [NM_145892.2](http://www.ncbi.nlm.nih.gov/nuccore/NM_145892.2) | CDS.11 | DELETION | c.1037_1042delCTGCCG | p.A347_A348del | E699 |
| 16 | 7726819 | 7726824 | 6 | CTGCCG | - | DEL | RBFOX1 | [CCDS10532.1](http://www.ncbi.nlm.nih.gov/CCDS/CcdsBrowse.cgi?REQUEST=CCDS&DATA=CCDS10532.1) | [NM_145893.2](http://www.ncbi.nlm.nih.gov/nuccore/NM_145893.2) | CDS.11 | DELETION | c.1037_1042delCTGCCG | p.A347_A348del | E699 |
| 16 | 7726819 | 7726824 | 6 | CTGCCG | - | DEL | RBFOX1 | [CCDS55983.1](http://www.ncbi.nlm.nih.gov/CCDS/CcdsBrowse.cgi?REQUEST=CCDS&DATA=CCDS55983.1) | [NM_018723.3](http://www.ncbi.nlm.nih.gov/nuccore/NM_018723.3) | CDS.12 | DELETION | c.974_979delCTGCCG | p.A326_A327del | E699 |
| 17 | 77090608 | 77090608 | 1 | G | T | SNP | RBFOX3 | [CCDS45805.1](http://www.ncbi.nlm.nih.gov/CCDS/CcdsBrowse.cgi?REQUEST=CCDS&DATA=CCDS45805.1) | [NM_001082575.1](http://www.ncbi.nlm.nih.gov/nuccore/NM_001082575.1) | CDS.14 | STOP_GAINED | c.861C>A | p.Y287* | E136 |

Hg19, detailed description of variants identified in *RBFOX1* and *RBFOX3*
